# The Impact of Small Extracellular Vesicles on Lymphoblast Trafficking across the Blood-Cerebrospinal Fluid Barrier In Vitro

**DOI:** 10.3390/ijms21155491

**Published:** 2020-07-31

**Authors:** Ulrike Erb, Julia Hikel, Svenja Meyer, Hiroshi Ishikawa, Thomas S. Worst, Katja Nitschke, Philipp Nuhn, Stefan Porubsky, Christel Weiss, Horst Schroten, Rüdiger Adam, Michael Karremann

**Affiliations:** 1 Department of Pediatrics, University Medical Center Mannheim, 68167 Mannheim, Germany; ulrike.erb@medma.uni-heidelberg.de (U.E.); julia.hikel@gmx.de (J.H.); svenja.meyer@medma.uni-heidelberg.de (S.M.); horst.schroten@umm.de (H.S.); ruediger.adam@umm.de (R.A.); 2 Laboratory of Clinical Regenerative Medicine, Department of Neurosurgery, Faculty of Medicine, University of Tsukuba, 1–1–1 Tennodai, Tsukuba, Ibaraki 305–8575, Japan; ishi-hiro.crm@md.tsukuba.ac.jp; 3 Department of Urology and Urosurgery, University Medical Center Mannheim, 68167 Mannheim, Germany; thomas.worst@umm.de (T.S.W.); katja.nitschke@umm.de (K.N.); Philipp.Nuhn@umm.de (P.N.); 4 Institute of Pathology, University Medical Center of the Johannes Gutenberg University Mainz, 55101 Mainz, Germany; Stefan.Porubsky@unimedizin-mainz.de; 5 Department of Medical Statistics and Biomathematics, University Medical Center Mannheim, 68167 Mannheim, Germany; christel.weiss@medma.uni-heidelberg.de

**Keywords:** choroid plexus, central nervous system infiltration, exosomes, pediatric acute lymphoblastic leukemia

## Abstract

Central nervous System (CNS) disease in pediatric acute lymphoblastic leukemia (ALL) is a major concern, but still, cellular mechanisms of CNS infiltration are elusive. The choroid plexus (CP) is a potential entry site, and, to some extent, invasion resembles CNS homing of lymphocytes during healthy state. Given exosomes may precondition target tissue, the present work aims to investigate if leukemia-derived exosomes contribute to a permissive phenotype of the blood-cerebrospinal fluid barrier (BCSFB). Leukemia-derived exosomes were isolated by ultracentrifugation from the cell lines SD-1, Nalm-6, and P12-Ichikawa (P12). Adhesion and uptake to CP epithelial cells and the significance on subsequent ALL transmigration across the barrier was studied in a human BCSFB in vitro model based on the HiBCPP cell line. The various cell lines markedly differed regarding exosome uptake to HiBCPP and biological significance. SD-1-derived exosomes associated to target cells unspecifically without detectable cellular effects. Whereas Nalm-6 and P12-derived exosomes incorporated by dynamin-dependent endocytosis, uptake in the latter could be diminished by integrin blocking. In addition, only P12-derived exosomes led to facilitated transmigration of the parental leukemia cells. In conclusion, we provide evidence that, to a varying extent, leukemia-derived exosomes may facilitate CNS invasion of ALL across the BCSFB without destruction of the barrier integrity.

## 1. Introduction

Survival of pediatric acute lymphoblastic leukemia (ALL) has dramatically improved over the last decades [1]. Yet still, treatment strategies to the central nervous system (CNS) compartment remain challenging and even prophylactic therapy in low risk patients may result in relevant sequelae [2]. On the other hand, most CNS relapses which occur in children are initially classified as CNS negative, indicating “undertreatment” in a subset of low-risk patients [3,4]. Therefore, predictive markers allowing to identify the personalized risk of CNS relapse more accurately are urgently warranted to further improve precise stratification of CNS directed therapy and prophylaxis in the future [5]. Identifying such markers is hampered by the fact that the nature of CNS invasion is not yet fully understood and mechanisms protecting lymphoblasts within the CNS compartment are diverse [6].

The present work will focus on the relevance of leukemia-derived exosomes in the process of CNS invasion. To date, it is still under debate, whether the transmigration into the CNS is a general trait of ALL blasts or restricted to specific subclones [4,7,8]. Distinct risk factors of CNS leukemia include hyperleukocytosis at diagnosis, T-cell phenotype, and several genetic alterations [9]. Additionally, various biological features affect the invasion potency of lymphoblasts, including the expression of cytokines, chemokines, growth factors, cell adhesion molecules, and matrix metalloproteases [3,4,10]. The role of small extracellular vesicles within this process is yet unclear [11]. Since their first description, multiple mechanisms have been unraveled by which tumor-derived exosomes affect the formation of CNS metastases in numerous malignancies [12,13]. These sum up, in the potential to modify tissues distant to the primary tumor site, and result in the formation of a “premetastatic niche”, facilitating circulating tumor cells to invade these tissues, and build new metastases, a mechanism that has also been published in CNS infiltration by hematologic malignancies [14,15]. In this regard, Kinjyo and colleagues have recently described the impact of leukemia-derived exosomes on lymphoblast transmigration across the blood-brain barrier (BBB) in pediatric ALL. In their model, leukemia-derived exosomes led to modulation of astrocytes and brain microvascular endothelial cells, resulting in upregulation of vascular endothelial growth factor A (VEGF-A) expression and sequential disruption of the BBB integrity [5]. Furthermore, exosomes may well be involved in molecular mechanisms facilitating survival and chemotherapy resistance of leukemic blasts within the CNS compartment, e.g., inducing cellular quiescence [16]. In this regard, varying microRNA expression profiles have been described in T-linage and B-cell precursor (BCP)-ALL, potentially contributing to the different clinical behavior of these ALL subtypes [17].

Multiple routes of leukemic blasts into the CNS compartment have been described [3,18]. However, despite the rarity of parenchymal metastases during initial stages, most in vitro studies applied models of the BBB. Despite its central role on immune cells traveling into the CNS [19], the BCSFB within the choroid plexus is yet under-recognized concerning the CNS infiltration by leukemic blasts [20]. Therefore, we investigated the relevance of leukemia-derived exosomes on ALL cell transmigration across the blood-cerebrospinal fluid barrier (BCSFB). Recently, we could establish a human in vitro model suitable to study the cellular interactions of leukemic blasts with the choroid plexus epithelial cells and demonstrate crossing of lymphoblasts by a transcellular and paracellular route [21]. Now, we provide evidence that leukemia-derived exosomes may facilitate this process of transmigration into the CNS compartment without destruction of the BCSFB integrity.

## 2. Results

### 2.1. Exosome Isolation

Extracellular vesicles released by the three leukemia cell lines SD-1 (BCP-ALL), Nalm-6 (BCP-ALL), and P12 (T-ALL) all presented with typical size of exosomes, and expressed exosomal markers including CD 63 and CD 81 (Supplementary Figure S1). The cell lines had proven capability to infiltrate the CNS in mouse models [22,23,24].

### 2.2. Time- and Dose-Dependent Uptake to HiBCPP Cells

**Figure 1:**
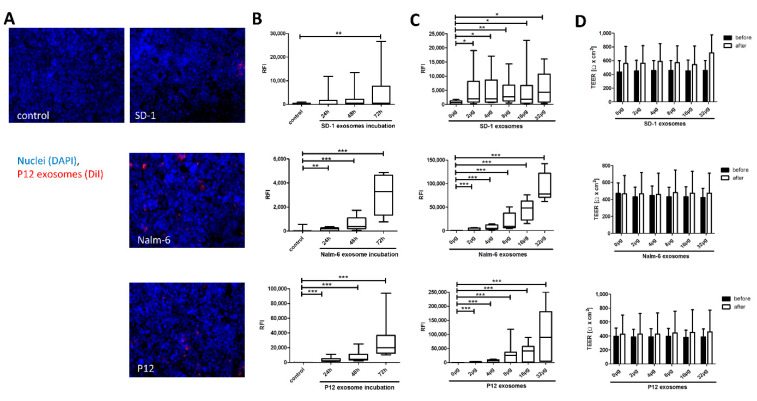
Time- and dose-dependent uptake/binding of acute lymphoblastic leukemia (ALL) cell-derived exosomes to blood-cerebrospinal fluid barrier (BCSFB) in vitro model. Fluorescently labeled exosomes were added onto the basolateral side of HiBCPP cells grown in inverted culture as indicated and uptaken/bound exosomes were quantified by fluorescent microscopy. Relative fluorescence intensity (RFI) was measured in 15 fields of view (20× objective). (**A**) Representative observation field of untreated control HiBCPP cells and HiBCPP cells incubated with DiIC18(3) labeled exosomes (red) (16 μg, 48 h). HiBCPP cells were visualized by nuclei staining (DAPI, blue). (**B**) Time-dependent uptake/binding of ALL-derived exosomes (16 μg per filter; SD-1, Nalm-6, and P12, respectively) for indicated time points from basolateral side, and (**C**) dose-dependent uptake/binding of ALL-derived exosomes (48 h; SD-1, Nalm-6, and P12, respectively) for indicated amounts per filter from basolateral side. (**D**) Barrier integrity following exosome incubation was determined by transepithelial electrical resistance (TEER) and showed no significant alterations compared to untreated control. All data shown are box plots with whiskers of at least 3 independent experiments performed in triplicates. * *p* < 0.05; ** *p* < 0.01; *** *p* < 0.001.

Exosome uptake to HiBCPP cells was determined for up to 72 h by fluorescent microscopy (Figure 1A), and differed markedly between the various cell lines: In SD-1-derived exosomes, uptake to plexus epithelial cells did not correlate with incubation time and exosome concentration. In contrast, in Nalm-6 and P12-derived exosomes, increasing incubation time and exosome concentration resulted in a significant increment of incorporation into HiBCPP cells (Figure 1B,C). Acid wash of incubated HiBCPP cells was performed to strip off exosomes adherent to the outer cell membrane to verify complete uptake to HIBCPP cells. Although relative fluorescence intensity (RFI) with acidic wash was slightly increased in Nalm-6-derived exosomes (median RFI 1954 versus 2910) and decreased in P12-derived exosomes (median RFI 1826 versus 685), none of these alterations was statistically significant, indicating that most (if not all) exosomes were completely incorporated to the HiBCPP target cells (Supplementary Figure S2).

Barrier function of HiBCPP cells was determined by both transepithelial electrical resistance (TEER) measurement before and after exosomal incubation and dextran-flux for the last 4 h of experiment, the latter to determine permeability to macromolecules. Neither did exosome incubation result in an increase of barrier tightness markers compared to untreated control, nor did exosome incubation lead to a disruption of barrier function (Figure 1D). Accordingly, viability of HiBCPP cells and expression of tight junction protein zonula occludens 1 (ZO-1) in the BCSFB in vitro model was not altered by exosome incubation throughout the experiments (Supplementary Figure S3).

### 2.3. Inhibition of Exosome Association and Uptake

**Figure 2:**
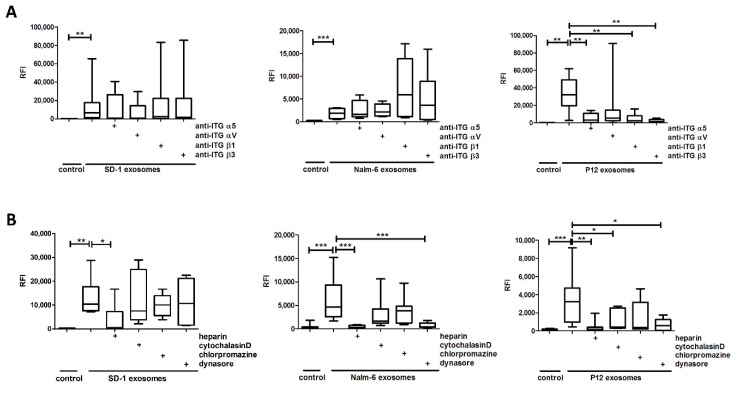
Mechanism of uptake/binding of acute lymphoblastic leukemia (ALL) cell-derived exosomes to blood-cerebrospinal fluid barrier (BCSFB) in vitro model. DiIC18(3) labeled exosomes (16 µg per filter) were added onto the basolateral side of HiBCPP cells grown in inverted culture and incubated for 48 h. The nuclei were stained with DAPI solution. Relative fluorescence intensity (RFI) of exosomes was quantified by fluorescent microscopy (15 fields of view, 20× objective). (**A**) Fluorescently labeled exosomes were pretreated with 4 µg/mL anti-integrin-antibody (anti-ITGα5; anti-ITGαV; anti-ITGβ1; anti-ITGβ3) for 30 min at 37 °C before incubation on HiBCPP cells. (**B**) HiBCPP cells were pretreated with chemical inhibitors (10 µg/mL heparin; 5 μg/mL cytochalasin D; 50 µM chlorpromazine; 80 µM dynasore) for 1 h at 37 °C before incubation. HiBCPP cells were washed with acidic PBS (pH = 2.5) after incubation to strip off adherend exosomes. Data shown are box plots with whiskers of at least 3 independent experiments performed in triplicates. * *p* < 0.05; ** *p* < 0.01; *** *p* < 0.001.

Next, we analyzed the integrin-dependent association of BCP-ALL and T-ALL-derived exosomes on BCSFB in vitro model using blocking antibodies to inhibit exosomal integrins, which bind to the Arginine, Glycine, and Aspartate motif (RGD-sequence) of extracellular matrix proteins, prior to incubation with HiBCPP cells. The adhesion of BCP-ALL-derived exosomes was not altered upon antibody blocking. Instead, the association of T-ALL P12-derived exosomes to HiBCPP cells was significantly diminished to proximately 6% to 10% when blocking exosomal integrin αV and β3 binding to fibronectin, vitronectin and osteopontin. Furthermore, the inhibition of integrin α5 and β1, also binding to vitronectin, decreased association to 12% to 14% compared to untreated exosomes (Figure 2A).

Moreover, we analyzed the mechanism of exosome uptake. Therefore, chemical inhibitors were applied to HiBCPP cells 1 h prior to incubation with fluorescently labeled exosomes. In addition, HiBCPP cells were washed with acidified phosphate buffered saline (PBS) to analyze only incorporated exosomes (Figure 2B). By heparin blocking the exosomal binding to cellular heparan sulfate proteoglycans, the adhesion and uptake of exosomes of all three ALL cell lines were reduced to 37%, 5% and 21% for SD1, Nalm-6 and P12 exosomes, respectively. Furthermore, using cytochalasin D to inhibit the actin-dependent micropinocytosis, the uptake of T-ALL P12 exosomes was reduced to approximately 62% compared to untreated HiBCPP cells. Nevertheless, the uptake of BCP-ALL SD1 and Nalm-6 exosomes was not significantly altered by cytochalasin D treatment. The inhibition of clathrin-dependent endocytosis via chlorpromazine did not affect the internalization rates of SD1, Nalm-6, or P12 exosomes. Instead, the dynamin-dependent endocytosis, which was inhibited by dynasore pre-incubation of HiBCPP cells, was diminished to 9% and 20% for Nalm-6 and P12 exosomes, respectively. Again, the uptake of SD1 exosomes was not inhibited.

### 2.4. Leukemia Cell Transmigration across the BCSFB

**Figure 3:**
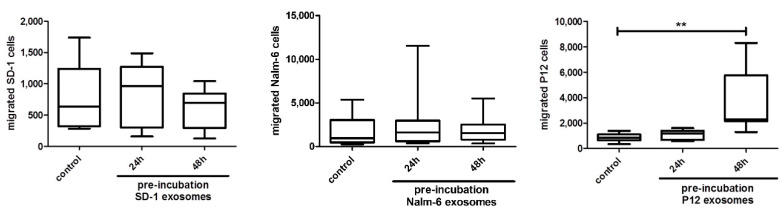
Exosomal pre-incubation enhances transmigration across blood-cerebrospinal fluid barrier (BCSFB) in vitro model of T-ALL cell line P12 but not of B-cell precursor (BCP)-ALL cell lines in time-dependent manner. CellTracker™ Green labeled ALL cells were added for 6 h onto the basolateral side of untreated HiBCPP cells or HiBCPP cells pre-incubated with 16 μg unlabeled exosomes for indicated time-points. C-X-C motif chemokine ligand 12 (CXCL12) (100 ng/mL) was used as chemoattractant. Migrated ALL cells (SD-1, Nalm-6, and P12, respectively) were quantified in the lower compartment by fluorescent microscopy (10 fields of view, 10× objective). Data shown are box plots with whiskers of at least 3 independent experiments performed in triplicates. * *p* < 0.05; ** *p* < 0.01; *** *p* < 0.001.

We analyzed the potential of leukemia-derived exosomes to facilitate transmigration of lymphoblasts across the BCSFB (Figure 3) We employed a human in vitro model and analyzed the transmigration rate of SD-1 (BCP-ALL), Nalm-6 (BCP-ALL), and P12 (T-ALL). Following incubation of HiBCPP with the respective leukemia-derived exosomes, transmigration during a 6 h period was significantly increased in P12 cells. Compared to an unstimulated HiBCPP control, transmigration rates were 1.4-fold (not significant) and 2.7-fold (*p* = 0.0009) increased, following exosome incubation of the BCSFB for 24 and 48 h, respectively.

In contrast, transmigration of SD-1 and Nalm-6 cells across the BCSFB during a 6 h period was not altered, when HiBCPP cells were pre-incubated with exosomes for 24 and 48 h. Both BCP-ALL cell lines presented with transmigration rates equal to those found across untreated plexus epithelium.

Again, exosome incubation of plexus epithelium did not result in an impairment of barrier function markers in any of the experiments. TEER values, dextran permeability, and immunofluorescent evaluation of tight junction protein expression over time were not altered significantly during the experiments.

## 3. Discussion

Many questions remain to be answered until CNS infiltration from pediatric acute lymphoblastic leukemia will be fully understood [3]. So far, several potential routes into the CNS compartment have been identified, and numerous molecular mechanisms have been described that drive infiltration to and survival within the CNS microenvironment [6,25]. In the present study, we investigated the role of leukemia-derived exosomes in CNS invasion of lymphoblasts across the BCSFB in a human in vitro model.

### 3.1. Exosome Conditioning Does Not Alter BCSFB Integrity

These small extracellular vesicles play a crucial role in tumor biology, and previous studies have unraveled their multifunctional role also in acute leukemias [14,15]. In this regard, Kinjyo and co-workers found an increased transmigration across human brain microvascular endothelial cells, representing the BBB, when leukemia-derived exosomes were added to their in vitro model [5]. Exosome uptake led to an upregulation of VEGF-A expression, resulting in a degradation of tight junction proteins and hence, increased permeability of the BBB. As a counterpart concerning the blood-CNS barriers, we now describe evidence that exosomes may also modulate leukemic CNS infiltration via the BCSFB, notably, without breakdown of the BCSFB following exosome uptake. Disruption of the brain barriers was a late event in a mouse model of CNS leukemia [5], correlating to in vivo findings where parenchymal metastases occur only occasionally during early stages [4]. However, subclinical CNS infiltration in pediatric ALL at diagnosis is more frequent than previously assumed, with most of these patients presenting without neurological signs of disturbed blood-CNS barriers [26,27]. Therefore, transmigration of lymphoblasts facilitated by exosome-associated conditioning of the barriers might well be due to more sophisticated mechanisms than just disruption of tight junctions and barrier breakdown, but e.g., by the induction of receptor expression or cytokine signaling [3,4,18]. In this regard, we could previously show transcellular migration of lymphoblasts across the choroid plexus epithelium, hence, disruption of tight junction proteins may not be necessary at all to cross the blood-CNS barriers and degradation of tight junction proteins may not be the only exosome-mediated pathway to facilitate lymphoblast invasion to the brain [21].

### 3.2. Mechanisms of Exosome Uptake to HiBCPP Cells

When adhesion to target cells could be inhibited by heparin in all cell lines indicating exosome binding to cellular heparan sulfate proteoglycans, exosome uptake and biological significance markedly differed between the various cell lines. Nalm-6 and P12-derived exosomes incorporated to HiBCPP cells mainly by dynamin-dependent endocytosis [28], without evidence of clathrin-dependent endocytosis and only a minor relevance of actin-dependent macropinocytosis (in P12 exosomes) [29,30]. In contrast, SD-1-derived exosomes seemed to associate to HiBCPP cells unspecifically, potentially fusing to the plasma membrane [31]. Given markedly different association to the BCSFB, a varying capability to precondition target tissue and finally foster CNS involvement across the BCSFB may be possible, in line with CNS metastases from solid tumors. In the latter, binding of tumor cell-derived exosomes to target tissue is highly regulated and dependent on exosomal expression of adhesion molecules binding to proteoglycans and extracellular matrix (ECM) proteins [32,33]. In this regard, exosomal integrin expression was crucial for metastasis distribution pattern of sub-lines of the breast cancer cell line MDA-MB-231, and brain metastases were driven by exosomes expressing integrin β3 [13]. Furthermore, the exosomal expression of integrin α5 and αV was associated to brain metastatic melanoma [34]. In pediatric ALL, various integrins expressed on lymphoblasts were linked to CNS disease and even associated to potential routes of infiltration [35]. Given that the receptor repertoire of exosomes resembles parental cells, it is yet to elucidate whether the above-mentioned findings may also be mediated via leukemia-derived exosomes. In this regard, reduced adhesion of P12-derived exosomes by blocking integrin α5, αV, β1, and β3 suggests a potential role of the integrin profile of ALL-derived exosomes on CNS disease. As blocking of integrins did not affect uptake of the BCP-ALL-derived exosomes, binding and uptake to HiBCPP cells may obviously follow multiple pathways and differ between exosomes from the various cell lines. Although we did not quantify the expression of the various integrins on exosomes, we hypothesize that these were prevalent on the extracellular vesicles, since all three ALL cell lines express the evaluated integrins [36,37,38].

### 3.3. Differential Relevance in BCP-ALL and T-ALL Cell Lines

The differential uptake to HiBCPP cells in BCP-ALL and T-ALL-derived exosomes may well be associated to a varying potency of conditioning the choroid plexus tissue. The present findings suggest a relevance of leukemia-derived exosomes on preconditioning the BCSFB to a permissive phenotype in the T-ALL cell line P12. Firstly, adhesion and uptake could be attributed to well-established mechanisms, and reduction of exosomal uptake by integrin blockade might point out to a choroid plexus epithelium specific process in P12-derived exosomes [13,31]. Secondly, exosome preconditioning facilitated leukemic blast transmigration across the BCSFB in the P12 T-ALL cell line, but not in the BCP-ALL cell lines. Hence, exosomes may differ to each other not only concerning association to HiBCPP, but also regarding the biological relevance following uptake [39]. Further research is warranted to verify such a hypothesis and elucidate the underlying molecular mechanisms of exosome preconditioning. Since both Nalm-6 and P12-derived exosomes were uptaken to the choroid plexus epithelial cells, but resulted in an increased transmigration rate of lymphoblasts only in the P12 cell line, a varying exosomal cargo might well explain the different behavior and (in part) finally contribute to the neurotropism of T-ALL invading the CNS across the BCSFB. In this regard, Almeida and co-workers compared microRNA expression in pediatric BCP-ALL versus T-ALL patients, and identified 16 differentially expressed microRNAs [17]. Targets of these microRNAs included proteins associated to CNS leukemia, e.g., Zeta Chain of T Cell Receptor Associated Protein Kinase 70 (ZAP70) and C-X-C modif chemokine ligand 12 (CXCL12) [6,40,41,42]. In addition, miR-151a-5b and miR-151b, downregulated in T-ALL patients, was also associated to CNS lymphoma [43]. Whether these microRNAs explain the differential transmigration following exosome incubation in the present study is to be elucidated. However, although the risk of CNS invasion is multifactorial and multiple routes to the CNS compartment have been described, identifying such specific exosomal content (including proteins or RNA fragments) associated to an increased BCSFB transmigration might serve as a potential prognostic and/or therapeutic target in the future [11,44]. To this end, in vitro models may serve as screening tools prior to verify hypotheses in animal studies.

### 3.4. Translational Implications

Lymphocyte homeostasis within the CNS compartment during healthy state is orchestrated by the BCSFB, and T-cells predominate the CSF [19,45]. Of note, pathways driving T-cell attraction to the CNS also underlie CNS infiltration of ALL, including the expression of C-C chemokine receptor type 7 (CCR7), P-selectin glycoprotein ligand 1 (PSGL-1), and integrins [35,46,47,48]. When the predilection of CNS involvement in T-ALL hijacks these pathways [18], the BCSFB within the choroid plexus might well represent a significant gate into the CNS. Several studies have addressed this potential entry site [7,20,21], but so far, have described only limited lymphoblast transmigration across the BCSFB [3,49]. However, pediatric ALL presents with only subtle symptoms in most patients, and time to diagnosis is often prolonged. Therefore, conditioning target tissue (e.g., the choroid plexus epithelium) by leukemia-derived exosomes and transmigration of lymphoblasts into the CNS compartment (by hematogenic route) may develop over several weeks. In this regard, the present findings of prolonged exosome uptake compared to studies on the BBB, associated to delayed increase of P12 transmigration not before 48 h from incubation, may argue for a yet under-recognized role of this entry site due to limited duration of experiments especially in T-ALL. Given the potential of exosomes to induce a pro-metastatic condition, efforts should be made to establish long-term models suitable to investigate the cellular interactions of lymphoblasts and their respective extracellular vesicles with the BCSFB.

### 3.5. Summary

Small extracellular vesicles play a crucial role in tumor dissemination and CNS infiltration may be facilitated by exosome-mediated transformation of target tissue not only in solid tumors, but also in leukemias [5,16]. Now, we could show that leukemia-derived exosomes enhance P12 transmigration across the BCSFB in vitro. In conclusion, exosomes may be involved in CNS homing, and the choroid plexus may serve as entry site to the CSF especially in T-ALL. To this end, further work is warranted to elucidate the exosome-induced molecular mechanisms driving CNS infiltration, and efforts are to be undertaken to establish long-term in vitro models to study this process over time. By identifying specific exosomal cargo and/or particular signaling, extracellular vesicles might serve as additional diagnostic markers in the future, and help to identify patients on increased risk of CNS involvement allowing further personalization of CNS directed treatment.

## 4. Materials and Methods

### 4.1. Cell Culture

The three ALL cell lines (SD-1, Nalm-6, and P12-Ichikawa) were chosen due to their capability to invade the CNS compartment in mouse models [22,23,24]. They were purchased from DSMZ (Braunschweig, Germany) and cultured under standard conditions (37 °C, 5% CO_2_, 95% humidity) in Dulbecco’s Modified Eagle Medium: Nutrient Mixture F-12 (DMEM/F12 (Ham)) supplemented with 10% fetal calf serum (FCS) (Thermo Fisher Scientific, Dreieich, Germany). HiBCPP cells [50] were cultured under standard conditions (37 °C, 5% CO_2_, 95% humidity) in DMEM/F12 (Ham) supplemented with 5 μg/mL insulin (Sigma Aldrich, Taufkirchen, Germany) and 10% FCS (Thermo Fisher Scientific, Dreieich, Dreieich, Germany).

### 4.2. Exosome Isolation

ALL cell lines were cultured in serum-free DMEM/F12 (Ham) under standard conditions (37 °C, 5% CO_2_, 95% humidity) for 48 h prior to isolation of ALL cell line-derived exosomes. Supernatant was cleared by differential centrifugation (200× *g* for 10 min, 300× *g* for 10 min, 1500× *g* for 20 min, 2500× *g* for 20 min, 4500× *g* for 30 min, 14,000× *g* for 30 min). Exosomes were pelleted down by ultracentrifugation (Sorvall WX Ultra 100 equipped with Sorvall SureSpin 630, Thermo Fisher Scientific, Dreieich, Germany) for 150 min at 100,000× *g*. Exosomes were resuspended in PBS (Thermo Fisher, Dreieich, Germany), filter-sterilized (0.22 μm, Sartorius, Göttingen, Germany) and stored at −80 °C. Where indicated, exosomes were stained with DiIC18(3) (Thermo Fisher, Dreieich, Germany) according to manufacturer’s instructions.

### 4.3. BCSFB In Vitro Model

HiBCPP cells were used as an in vitro model of the BCSFB as previously described [21]. In brief, 7.5–9 × 10^4^ HiBCPP cells were seeded on the bottom side of an inverted 24-well-filter insert with a pore size of 5 μm (Sarstedt, Nümbrecht, Germany) in DMEM/F12 (Ham) supplemented with 5 μg/mL insulin and 10% FCS (Thermo Fisher, Dreieich, Germany) and adhered for 24 h under standard conditions (37 °C, 5% CO_2_, 95% humidity.) Subsequently, filter inserts were placed in standard orientation and HiBCPP cells were cultured until TEER reached at least 70 Ωcm^2^ (epithelial voltohmmeter, Millicell ERS STX-1, Merck Millipore, Darmstadt, Germany) and medium was replaced to DMEM/F12 (Ham) supplemented with 5 μg/mL insulin and 1% FCS overnight, leading to tighter barrier properties. HiBCPP cells with a TEER between 250–800 Ωcm^2^ were used for experiments.

### 4.4. Uptake of Exosomes by BCSFB In Vitro Model

Fluorescently labeled exosomes were added onto the basolateral side of HiBCPP cells in the upper compartment of filter inserts and incubated under standard conditions (37 °C, 5% CO_2_, 95% humidity) as indicated. HiBCPP cells of BSCFB in vitro model were washed 4 times with PBS or where indicated with acidic PBS (pH = 2.5) after first round of PBS wash, and two times more with PBS afterwards. Cells were fixed with 4% formaldehyde/PBS for 10 min at room temperature (RT). Filters were subsequently cut out and cells were permeabilized for 10 min with 0.1% Triton X-100/1% bovine serum albumin (BSA)/PBS. After washing two times with 1% BSA/PBS, filters were blocked for 20 min in 1% BSA/PBS and stained with 4’,6-Diamidino-2-Phenylindole (DAPI) solution (1.5:55,000, Invitrogen, Carlsbad, CA, USA) to stain the nuclei for 10 min at RT. Filters were washed, mounted with Prolong Gold antifade reagent (Invitrogen, Carlsbad, CA, USA). Exosomes were quantified by fluorescent microscopy (15 fields of view, 20× objective, Axio Observer.Z1, ZEN2 pro software, blue edition, Carl Zeiss, Oberkochen, Germany). The barrier integrity was observed throughout the experiments. TEER was measured at the beginning and end of each experiment and dextran flux (Dextran TexasRed, 100 μg/mL, Invitrogen, Carlsbad, CA, USA) was monitored for 4 h. The fluorescence of Dextran TexasRed in the lower compartment was measurement in Tecan Infinite M200 Multiwell reader (Tecan, Männedorf, Switzerland) afterwards. Where indicated, the HiBCPP cells of BCSFB in vitro model were pretreated with chemical inhibitors (Dynasore, 80 μM, Merck Millipore, Darmstadt, Germany; Heparin, 10 μg/mL, Sigma Aldrich, Steinheim, Germany; Cytochalasin D, 5 μg/mL, Sigma Aldrich, Steinheim, Germany; Chlorpromazine, 50 μM, Sigma Aldrich, Steinheim, Germany) for 1 h. After removal of inhibitors, 16 μg of fluorescently labeled exosomes were added onto the basolateral side of HiBCPP cells and incubated for 48 h. Where indicated, the fluorescently labeled exosomes were pretreated with 4 μg/mL anti-integrin-antibody (anti-ITGα5, Immunotools, Friesoythe, Germany; anti-ITGαV Immunotools, Friesoythe, Germany; anti-ITGβ1, Merck Millipore, Darmstadt, Germany; anti-ITGβ3, BD Bioscience, San Jose, CA, USA) for 30 min at 37 °C before incubation for 48 h on HiBCPP cells.

### 4.5. Transmigration Assay

ALL cells were labeled with CellTrackerTM Green CMFDA Dye (1.5 μM, Invitrogen, Carlsbad, CA, USA) according to manufacturer’s instructions prior to transmigration experiments. 4 × 10^5^ labeled ALL cells were placed in the upper compartment of BCSFB in vitro model for 6 h at 37 °C in DMEM/F12 (Ham) supplemented with 0.5% BSA. CXCL12 (100 ng/mL, Peprotech, Cranbury, NJ, USA) was used in the lower compartment as chemoattractant as previously established [21]. The barrier integrity was measured at the beginning and end of each experiment by TEER.

Migrated ALL cells were quantified in the lower compartment by fluorescent microscopy (10 fields of view, 10× objective, Axio Observer.Z1, ZEN2 pro software, blue edition, Carl Zeiss, Oberkochen, Germany). Where indicated, BCSFB in vitro model of HiBCPP cells were pretreated with 16 μg of unlabeled exosomes per filter for up to 48 h prior to transmigration.

### 4.6. Western Blotting

ALL cells were lysed in modified radioimmunoprecipitation assay (RIPA) buffer (1× RIPA lysis buffer, 50 mM NaF, 1 mM Na_3_VO_4_, 1 mM phenylmethylsulfonyl fluoride (PMSF), protease inhibitor cocktail). Protein concentration of cell lysate or exosomes was determined by the Lowry method (DC Protein Assay, BioRad, Hercules, CA, USA) according to the manufacturer’s instructions. 20 μg of whole protein cell lysate or exosomes were boiled in 1× NuPage lithium dodecyl sulfate (LDS) Sample Buffer (Life Technologies, Carlsbad, CA, USA) for 10 min at 98 °C and resolved in 4–12% Bis Tris NuPage^®^ gels (Invitrogen, Carlsbad, CA, USA). After transfer onto nitrocellulose membranes (0.45 μm, BioRad, Hercules, CA, USA), proteins were detected with the following antibodies (1 μg/mL each): anti-CD63 (BD Bioscience, San Jose, CA, USA), anti-CD81 (BioLegend, San Diego, CA, USA), anti-ALIX (BioLegend, San Diego, CA, USA) and anti-β-actin (Life Technologies, Carlsbad, CA, USA) and visualized using Immobilon Western Chemiluminiscent HRP Substrate (Millipore, Schwalbach, Germany).

### 4.7. Viability Testing

For viability analysis, HiBCPP cells of BCSFB in vitro model were stained using a Live/Dead assay (Invitrogen, Carlsbad, CA, USA) according to manufacturer’s instructions and visualized by fluorescent microscopy (10× objective, Axio Observer.Z1, ZEN2 pro software, blue edition, Carl Zeiss, Oberkochen, Germany).

### 4.8. Immunofluorescence Staining

HiBCPP cells of BSCFB in vitro model were washed 4 times with PBS or where indicated with acidic PBS (pH = 2.5) after first round of PBS wash, and two times more with PBS afterwards. Cells were fixed with 4% formaldehyde/PBS for 10 min at RT. Filters were subsequently cut out and cells were permeabilized for 10 min with 0.1% Triton X-100/1% BSA/PBS. After washing two times with 1% BSA/PBS, filters were blocked for 20 min in 1% BSA/PBS and stained with primary antibody (anti-ZO-1; 1:250, Invitrogen, Carlsbad, CA, USA) overnight at 4 °C. After washing the filters three times with 1% BSA/PBS the following day, filters were incubated with secondary antibody (anti-rabbit Alexa Fluor 594, 1:250, Invitrogen, Carlsbad, CA, USA) for 2 h at RT and washed three times with 1% BSA/PBS. Cells were incubated with DAPI solution (1.5:55,000, Invitrogen, Carlsbad, CA, USA) to stain the nuclei and with Phalloidin Alexa Fluor 660 (1:250, Invitrogen, Carlsbad, CA, USA) for actin cytoskeleton for 10 min at RT. Filters were washed, mounted with Prolong Gold antifade reagent (Invitrogen, Carlsbad, CA, USA) and analyzed by fluorescent microscopy (63× objective, Axio Observer.Z1 with Apotome, ZEN2 pro software, blue edition, Carl Zeiss, Oberkochen, Germany).

### 4.9. Nanoparticle Tracking Analysis (NTA)

For NTA using ZetaView (Particle Metrix, Meerbusch, Germany), 1 µL of exosomes were diluted in sterile-filtered PBS 1:1000 and ZetaView settings were adjusted to sensitivity 80%, shutter 100, 11 positions, and 2 cycles.

### 4.10. Electon Microscopy

Native exosome suspension (10 µL) was placed on a 200 mesh copper grid with a formvar carbon support film (Plano, Wetzlar, Germany). After 10 min at room temperature, the fluid was deducted by filtering paper followed by 2 washing steps with distilled water. The grid was covered by 10 µL 1% uranyl acetate (Serva, Heidelberg, Germany) for 5 min followed by 2 washing steps with distilled water. Pictures were taken on transmission electron microscope JEM-1400 (Joel, Freising, Germany).

### 4.11. Statistics

Statistical analyses were conducted using SAS Software, release 9.4 (SAS Institute Inc., Cary, NC, USA). The Kruskal-Wallis test was used followed by the non-parametric Wilcoxon Rank-Sum Test for pairwise comparisons and exact *p* values have been requested. Box plots (min-max) were used to display relative fluorescence intensity (RFI) and transmigration, TEER and dextran flux are represented as mean ± SD. A test result was considered statistically significant for *p* values below 0.05.

## Supplementary Materials

Supplementary materials can be found at https://www.mdpi.com/1422-0067/21/15/5491/s1.

## Abbreviations

ALL	acute lymphoblastic leukemia
BBB	blood-brain barrier
BCP-ALL	B-cell precursor acute lymphoblastic leukemia
BCSFB	blood-cerebrospinal fluid barrier
CCR7	C-C chemokine receptor type 7
CNS	central nervous system
CP	choroid plexus
ECM	extracellular matrix
ITG	integrin
NTA	nanoparticle tracking analysis
PSGL-1	P-selectin glycoprotein ligand 1
RT	room temperature
RFI	relative fluorescence intensity
T-ALL	T-linage acute lymphoblastic leukemia
TEER	transepithelial electrical resistance
