# Supplementary material for: The Impact of Small Extracellular Vesicles on Lymphoblast Trafficking across the Blood-Cerebrospinal Fluid Barrier In Vitro
Source: Int J Mol Sci. 2020 Jul 31;21(15):5491. doi: 10.3390/ijms21155491 (PMC7432056; doi:10.3390/ijms21155491)
Supplement: Supplementary file 1 [file ijms-21-05491-s001.pdf]

Supplementary Figures

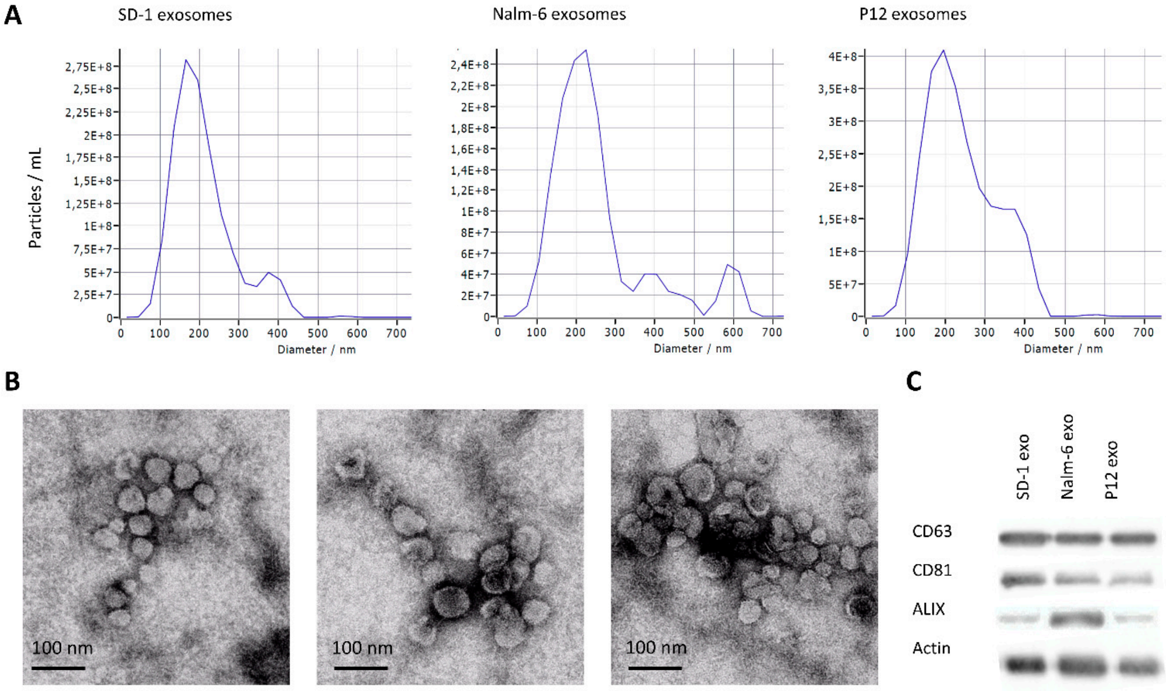

Supplementary Figure S1. Characterization of acute lymphoblastic leukemia (ALL) cell-derived exosomes. (**A**) Full size profiles of isolated exosomes of B-cell precursor (BCP)-ALL cell line SD-1 and Nalm-6 as well as T-ALL cell line P12 are shown (ZetaView®). (**B**) Transmission electron microscopy images from the cell culture supernatants showing groups of exosomes with a diameter from 30 to 80 nm. Bar indicates 100 nm. (**C**) Western blot analysis showed expression of exosomal protein markers CD63, CD81 and Alix as well as β-actin as loading control.

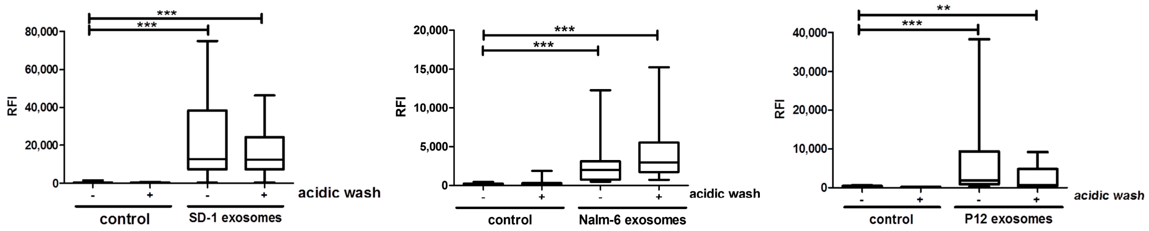

Supplementary Figure S2. Quantification of adherent and uptaken exosomes (- acidic wash) and uptaken exosomes (+ acidic wash). Fluorescently labeled exosomes (16 µg per filter; SD-1, Nalm-6, and P12, respectively) were added onto the basolateral side of HiBCPP cells grown in inverted culture for 48 h. Thereafter HiBCPP cells were washed with PBS or where indicated with acidic PBS (pH = 2.5) to strip off only adherend exosomes. The nuclei were stained with DAPI solution. Relative fluorescence intensity (RFI) of exosomes was quantified by fluorescent microscopy (15 fields of view, 20× objective). Data shown are box plots with whiskers of at least 3 independent experiments performed in triplicates. * $p < 0.05$; ** $p < 0.01$; *** $p < 0.001$.

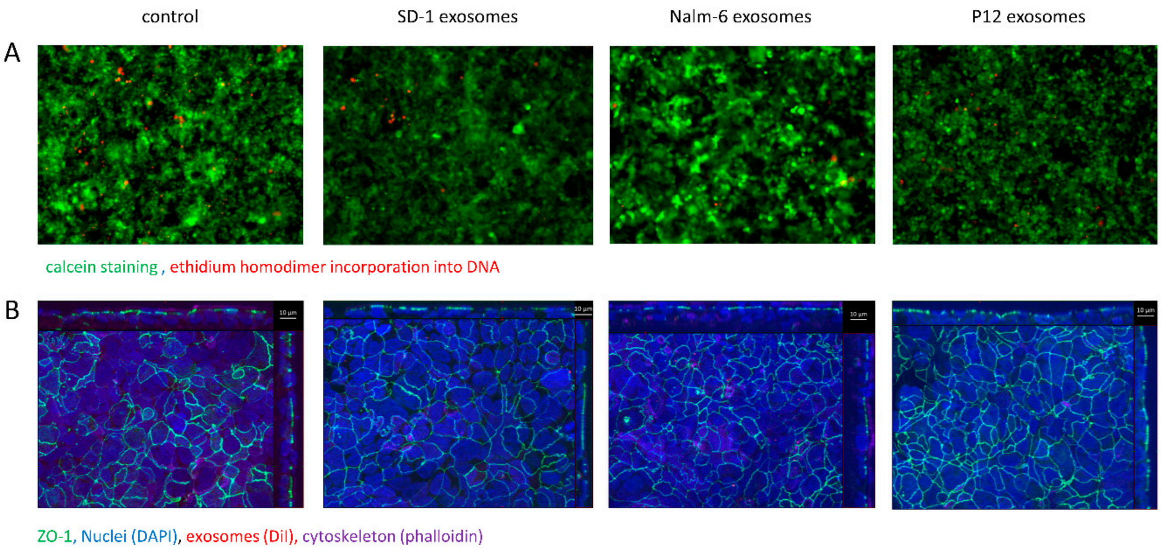

Supplementary Figure S3. The viability and expression of tight junction protein ZO-1 in blood-cerebrospinal fluid barrier (BCSFB) in vitro model are not disturbed by acute lymphoblastic leukemia (ALL)-derived exosome uptake/ binding. (**A**) Viability was comparable with untreated control HiBCPP cells and HiBCPP cells incubated with unlabeled exosomes (16 µg) for 48 h. Red staining indicates apoptotic HiBCPP epithelial cells according to the protocol of live/dead assay showing vital cells by calcein staining (green) and apoptotic cells by ethidium homodimer incorporation into DNA (red). Representative fluorescent microscopic images are shown. (**B**) Tight junctions of untreated control HiBCPP cells or HiBCPP cells incubated with DiIC18(3) labeled exosomes (16 µg, 48 h, red) were visualized with anti-ZO-1 antibody (green). Actin cytoskeleton is stained with phalloidin (magenta) and nuclei with DAPI (blue). Representative fluorescent microscopic images of maximum-intensity projection of z-stacks and corresponding orthogonal projections are shown. Scale bar: 10 µm.
